# Patient and Clinician Perspectives on Alert-Based Remote Monitoring–First Care for Cardiovascular Implantable Electronic Devices: Semistructured Interview Study Within the Veterans Health Administration

**DOI:** 10.2196/66215

**Published:** 2025-04-04

**Authors:** Allison Kratka, Thomas L Rotering, Scott Munson, Merritt H Raitt, Mary A Whooley, Sanket S Dhruva

**Affiliations:** 1 University of California, San Francisco School of Medicine, San Francisco, CA, United States; 2 Department of Medicine, San Francisco VA Health Care System, 4150 Clement St, San Francisco, CA, 94121, United States; 3 Section of Cardiology, Veterans Affairs Portland Health Care System, Portland, OR, United States; 4 Knight Cardiovascular Institute, Oregon Health & Science University, Portland, OR, United States

**Keywords:** cardiovascular implantable electronic device, CIED, remote monitoring, RM, alert-based monitoring, remote monitoring–first care, patient perspectives, clinician perspectives, veteran, pacemaker, implantable cardioverter-defibrillator, mobile phone

## Abstract

**Background:**

Patients with cardiovascular implantable electronic devices (CIEDs) typically attend in-person CIED clinic visits at least annually, paired with remote monitoring (RM). As the CIED data available through in-person CIED clinic visits and RM are nearly identical, the 2023 Heart Rhythm Society expert consensus statement introduced “alert-based RM,” an RM-first approach where patients with CIEDs that are consistently and continuously connected to RM, in the absence of recent alerts and other cardiac comorbidities, could attend in-person CIED clinic visits every 24 months or ultimately only as clinically prompted by actionable events identified on RM. However, there is no published information about patient and clinician perspectives on barriers and facilitators to such an RM-first care model.

**Objective:**

We aimed to understand patient and clinician perspectives about an RM-first care model for CIED care.

**Methods:**

We interviewed 40 rural veteran patients who were experienced with RM with CIEDs and 22 CIED clinicians who were experienced in using RM regarding barriers and facilitators to an RM-first care model. We conducted a reflexive thematic analysis of interviews. Two authors familiarized themselves with the dataset and generated separate codebooks based on the interview guides and inductively coded notes. These 2 authors met and reviewed each other’s codes, sought additional author input, and resolved differences before 1 author coded the remaining interviews and developed candidate themes. These themes were refined, named, and supported with quotations.

**Results:**

Patients expressed interest in an RM-first approach, to reduce the burden of long travel times, sometimes in inclement weather, and to enable clinicians to provide care for other patients. However, many preferred routine in-person visits; reasons included a skepticism of the capabilities of RM, a sense that in-person visits provided superior care, and enjoyment of in-person patient-clinician relationships. Clinicians were interested in RM-first care, especially for stable, RM-adherent patients who were not device-dependent. Clinicians most frequently cited the benefit of reducing patient travel burden as well as optimizing clinic space and time to focus on other care such as reviewing routine RM transmissions, but also noted barriers including lack of in-person assessment, patient-perceived diminution of the patient-clinician relationship, possible loss to follow-up, and technological difficulties. Clinicians felt that an RM-first care model should be evaluated for success based on patient satisfaction and assessment of timely addressing of rhythm issues to prevent adverse outcomes. Most clinicians believed that RM-first care represented the future of CIED care.

**Conclusions:**

Both patients and CIED clinicians interviewed who were experienced in using RM were open to an RM-first care model that reduces in-person visits but reported some barriers to solely relying on RM and possible diminution of the patient-clinician relationship. Implementation of new RM recommendations will require attention to these perceptions and prioritization of patient-centered approaches.

## Introduction

Remote monitoring (RM) is the standard of care for patients with cardiovascular implantable electronic devices (CIED; pacemaker or implantable cardioverter-defibrillator [ICD]) [1,2]. RM involves sending CIED data from a patient’s residence via a transmitter or smartphone app. Routine transmissions are usually sent every 90 days and can also be patient- or alert-initiated. RM is a Class 1, Level of Evidence A, professional society recommendation because of its many clinical outcome benefits [1,2]. These include reduced mortality [3-5], fewer hospitalizations [3,6], fewer inappropriate ICD shocks [7], as well as high patient satisfaction [8].

In addition to RM, CIEDs can also be checked in person; traditionally, patients attend routine in-person clinic visits at least annually [1]. However, because nearly all of the same CIED-related data can be obtained via RM, an alternative would be to end in-person visits completely if patients were consistently and continuously connected to RM, with in-person evaluations only when needed for clinically actionable reasons, such as CIED reprogramming [2].

The 2023 Heart Rhythm Society (HRS) expert consensus statement on practical management of the remote device clinic introduced such a novel care model, “alert-based RM,” in which patients with CIEDs that are consistently and continuously connected to RM, in the absence of recent alerts or other cardiac comorbidity, could attend in-person CIED clinic visits every 24 months (class 2a recommendation) [2]. This statement is supported by multiple randomized, controlled trials that have demonstrated no difference in cardiovascular events [2,9-11] while reducing in-person visits, loss to follow-up, staff workload, and costs of care [9-11].

Additionally, the professional society expert consensus discussed the possibility of ending all routine in-person visits, given that these visits may be “low-value” because most conclude that the CIED is working properly [2]. In-person visits would occur only as clinically prompted by actionable events identified on RM. Such an RM-first care model, where patients have routine in-person visits every 2 years, or even only as needed, if they remain consistently and continuously connected could be especially helpful for the Veterans Health Administration (VHA) patient population, because approximately 40% of veterans with CIEDs who participate in RM live in a rural area [12] (defined as a land area outside of a census tract with ≥30% of the population residing in an urbanized area as defined by the Census Bureau) [13] and often have long travel times to clinic visits.

Despite these potential advantages and the HRS recommendation supported by multiple randomized controlled trials, patient and clinician perspectives on this new care model have not been studied. To understand barriers and facilitators to implementation, we conducted a mixed methods evaluation to explore the perspectives of device clinicians and veterans with CIEDs on an RM-first care model.

## Methods

### Interview Guide and Survey Development

One semistructured interview guide for veteran patients and one for clinicians (Multimedia Appendix 1) was developed by the investigator team using the updated Consolidated Framework for Implementation Research [14]. The veteran interview guide was developed based on a prior veteran survey about RM [15] and revised with input from the Rural Colorado Veteran Research Engagement board. The clinician interview guide was developed through an iterative process with input solicited from practicing VHA cardiologists and the incorporation of concepts from new HRS recommendations [2].

Both interview guides sought to understand barriers and facilitators to an “RM-first strategy,” defined as in-person CIED clinic visits only if clinically prompted among patients engaged in RM. Patients were informed that similar data were obtained through RM as in-person visits; they may need in-person visits for abnormalities identified on remote transmissions; they could still contact their device clinic; and their other visits, such as with primary care, would continue. Patients were asked about the travel burden to VHA, how their care may have changed during the COVID-19 pandemic, and any concerns about reducing routine in-person CIED clinic visits. Device clinicians were asked about the benefits and barriers to this new care model, and how this may change their practice flow. A 23-item Qualtrics survey was also administered to gather professional and demographic data as well as preinterview information about clinician impressions of RM-first care (Multimedia Appendix 1). Specifically, this survey asked clinicians how often they conducted routine evaluations for patients with CIEDs, stratified by adherent and nonadherent patients, and what clinicians did when patients did not want to schedule routine in-person CIED checks or missed an in-person CIED check. This survey also asked clinicians about the anticipated benefits and concerns of an RM-first strategy, how effective that it would be concerning cardiovascular outcomes, and if such a strategy would help their clinic.

Of note, partway through the clinician interview process, the draft 2023 HRS expert consensus was released [2], introducing an “alert-based care” model, similar to RM-first care. Therefore, the interview guide was then adapted to solicit feedback about this recommendation. For the veteran interviews, a question was added about the veteran’s view of the new recommendations.

This was a quality improvement project conducted in partnership with the VHA Measurement Science Quality Enhancement Research Initiative and the VHA National Cardiac Device Surveillance Program.

### Study Population and Contact Process

Veterans were eligible for interview inclusion if they had a CIED, were completely adherent to RM in the past 400 days (which means that they had sent a remote transmission covering this timeframe), [12] and lived in a rural area. Introductory letters were sent to 100 randomly selected veterans meeting these criteria (since these participants did not know the project team), 91 of whom were then contacted at least once via a telephone connection to Microsoft Teams. The letter described the study background and objectives as well as topics that would be covered by a named VHA staff member (SM). Up to 3 contact attempts were made, with a message left for each unanswered attempt.

A purposive sample of VHA CIED clinic-focused clinicians who had been interviewed for a prior project about best practices to support RM adherence were contacted for interview [16]. An introductory email described this study’s background, objectives, and potential changes that may result from findings as well as information about the project team and funding source. Snowball sampling was then used, asking these clinicians to recommend colleagues at their device clinic. Finally, purposive sampling was used to contact clinicians caring for a high proportion of veterans living in rural areas to more adequately represent rural clinician perspectives.

### Interview Process

Informed consent was obtained before recording all interviews, which were conducted on and recorded using Microsoft Teams. Between November 2022 and February 2023, a total of 40 veterans were interviewed by coauthor SM (BS, male, qualitative researcher), with each of these 40 individual interviews lasting 5‐15 minutes in length and some attended by coauthors TLR (MPH, male, public health researcher) and SSD (MD, MHS, male, cardiologist). Between November 2022 and February 2023, a total of 22 clinician interviews between 30‐60 minutes were conducted by TLR, with some attended by SSD. Field notes were taken during both sets of interviews to summarize key points and supplemented with transcribed interview recordings to ensure accuracy. There were no repeat interviews.

### Qualitative Data Analysis

Reflexive thematic analysis [17,18] of interview field notes and transcripts was used to elucidate veteran and clinician views about RM-first care.

First, authors AK (MD, female, cardiology fellow) and TLR familiarized themselves with the dataset by reading the field notes and transcripts, making notes about the overall findings within both sets of interviews (veteran and clinician) and reflecting on their experiences in the direct care of patients with CIEDs (AK) and research and quality improvement efforts for care of patients with CIEDs (TLR). Next, the authors generated separate codebooks based on the domains of the distinct interview guides. For veteran interviews, AK and TLR independently coded 6 distinct interview notes, which involved generating additional codes identified inductively, for the goal of reflexivity. These 2 authors then met and reviewed each other’s codes, sought SSD’s input, and resolved any differences by consensus, creating 1 final codebook. AK then coded the remaining interviews and developed candidate themes, supporting each theme based on coded data and direct quotations. AK’s candidate themes were intentionally broad. TLR and SSD reviewed these themes with AK against the coded data, leading to refining and then naming these themes. Finally, AK wrote the analytic narrative and supported these themes with quotations directly from the veteran interviews to describe veteran perspectives. Coauthor SSD provided iterative feedback on several versions of the analytic narrative to improve clarity and increase confirmability.

For clinician interviews, AK and TLR first independently coded 3 distinct interview notes, which involved generating additional codes identified inductively. These 2 authors then reviewed each other’s codes and resolved any differences by consensus. AK then coded the remaining interviews. The authors used the same process as described above for thematic generation, refinement, and naming. AK wrote the analytic narrative, which is presented in the Results section of this paper, and supported these themes with quotations directly from the interviews. We conducted both clinician and patient interviews until reaching thematic saturation on two criteria, (1) no new concepts were identified in iterative analysis interviews (code frequency counts) and (2) there was consistent repetition among interviewee responses without any new information being added to existing codes (code meaning) [19,20]. The number of interviews that we conducted with both our population of veterans and Veterans Affairs (VA) clinicians exceeded the number (n=17) found in recent empiric studies [20].

Atlas.ti 23 (ATLAS.ti Scientific Software Development GmbH), a qualitative analysis software, was used to organize and apply analytic codes.

### Ethical Considerations

This work was conducted as a quality improvement project and not human subjects research. Per the Department of Veterans Affairs Office of Research & Development Program Guide: 1200.21, “VHA (Veterans Health Administration) Operations Activities That May Constitute Research,” data were collected as part of a quality improvement study to assess and improve the quality of RM care for veterans with CIEDs and did not require institutional review board approval. Veteran and clinician participants were informed at study enrollment that responses would be anonymized, and verbal consent to recording was acquired before each interview. No compensation was provided. Study data were deidentified and stored in a secure, encrypted VA database.

## Results

### Veteran Interviews

#### Overview

**Figure 1.**
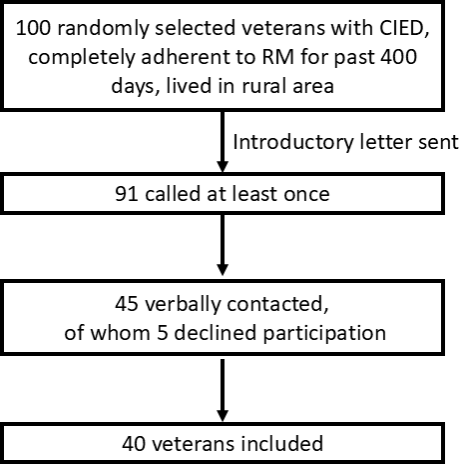
Flow diagram for veteran contact. CIED: cardiovascular implantable electronic device; RM: remote monitoring.

Among the 100 veterans who were initially mailed a letter to request participation, for patient sex, 97 (97%) were male and 3 (3%) were female; for patient race, 2 (2%) were American Indian or Alaska Native, 7 (7%) were Black or African-American, 3 (3%) were Native Hawaiian or other Pacific Islander, 81 (81%) were White, 5 (5%) declined to answer, and 1 (1%) was unknown; and for patient ethnicity, 1 (1%) was Hispanic or Latino, 96 (96%) were not Hispanic or Latino, 1 (1%) declined to answer, and 2 (2%) were unknown. Of 45 veterans contacted, 40 agreed to an interview (5 declined; Figure 1). The mean patient age was 77.6 (SD 8.9) years and all 40 were male (Table 1).

For their current care, most patients reported attending routine in-person visits to have their CIED checked (Table 1), usually every 6‐12 (range 2‐12) months. Many patients bundled other in-person VHA visits for convenience. Most patients did not think the COVID-19 pandemic had significantly changed their current CIED care.

When asked about an RM-first care model, 4 veterans preferred RM-first, 16 were amenable, 2 had no preference, and 18 did not want it. When asked what feedback they would prefer in an RM-first care model, few veterans wanted to know only when there was a problem, whereas more wanted feedback regarding successful or normal transmissions. The themes of barriers and facilitators to RM-first care described by veterans are in Table 2.

**Table 1.** Characteristics of veterans interviewed (n=40).

		Veterans interviewed
Age (years), mean (SD)		77.6 (8.9)
Gender, n (%)		
	Male	40 (100)
	Female	0 (0)
Race, n (%)		
	American Indian or Alaska Native	1 (2)
	Black or African American	2 (5)
	Native Hawaiian or other Pacific Islander	1 (2)
	White	35 (88)
	Declined to answer	1 (2)
Ethnicity, n (%)		
	Hispanic or Latino	0
	Not Hispanic or Latino	39 (98)
	Unknown	1 (2)
Type of device, n (%)		
	Implantable cardioverter-defibrillator	18 (45)
	Pacemaker	22 (55)
	Wireless-capable device^a^	34 (85)
Attended an in-person device clinic visit in the past year, n (%)		
	Yes	23 (58)
	No	17 (43)
Attended a telephone device clinic visit in the past year, n (%)		
	Yes	28 (70)
	No	12 (30)
Attended a VA^b^ Video Connect device clinic visit in the past year, n (%)		
	Yes	3 (8)
	No	37 (93)
Travel time to the VA (time for 1-way trip), n (%)		
	Less than 1 h	17 (42)
	1‐2 h	15 (38)
	2‐3 h	6 (15)
	More than 4 h	2 (5)
Patient-reported frequency of in-person device clinic visits, n (%)		
	Every 2‐3 weeks	1 (2)
	Every 2 months	2 (5)
	Every 3‐4 months	6 (15)
	Every 6 months	13 (32)
	>6 months and <1 year	3 (8)
	Every year	13 (32)
	Not available	2 (5)

a For context only, the 6 devices that were not wireless-capable were all pacemakers.

b VA: Veterans Affairs.

**Table 2.** Themes of barriers and facilitators to remote monitoring-first care.

Barriers	Facilitators
Veterans	
Importance of in-person care	Travel burden
Concerns about the adequacy of RM^a^ technology for care	Weather-related concerns
Loss of clinician-patient relationship	Comfort with technology
N/A^b^	Reducing the burden on the VHA^c^ device clinic
Clinicians	
Benefits of routine in-person assessment	Reduced veteran travel burden
Reducing veteran contact with VHA	Optimization of clinic space and clinic staff time
Clinic operations-related changes	More time to review routine transmissions and improve RM adherence
Technology and technological difficulties for veterans and clinicians	No concern about relative value units

a RM: remote monitoring.

b N/A: not applicable.

c VHA: Veterans Health Administration.

#### Barriers to RM-First Care

##### Importance of In-Person Care

Many patients who were not amenable to RM-first care believed that in-person evaluations provided more valuable information and essential care that could not be obtained another way. As one veteran stated,

*In person… they take a lot of recordings and stuff when they check the defibrillator… I think that it is [more accurate].*

##### Concerns About Adequacy of RM Technology for Care

Many veterans expressed concerns about the adequacy of RM technology for care. For some, this was based on a lack of comfort and sometimes a lack of confidence in RM technology or a belief that they needed more care because they had serious cardiac conditions.

*[Remote monitoring] is a good idea if we can understand what to do with the electronics… That is a little difficult for us.*

Some of these concerns may stem from an expressed lack of information about the capabilities of RM, what parameters are obtained from RM, and what clinicians do with that information.

*I’m not sure how they can check my [device] with the online system that I have…I don’t see how they would do it virtually, because they usually have to put a wand over the pacemaker to check its function.*

##### Loss of Clinician-Patient Relationship

A few patients noted that the loss of their relationship with their clinician would be a barrier to an RM-first care model.

*I actually look forward to the patient to doctor type meetings… there’s something to be said about personal visits.*

### Benefits of RM-First Care

#### Travel Burden and Weather-Related Concerns

Many veterans noted less time and cost burden would be required for travel to their VHA facility. For a few patients, this was related to poor mobility.

*It saves me 100 miles of driving, and if we can accomplish the same thing, I think that would be a lot better.*

*I don’t have to spend an hour on the highway and save on gas too.*

For some veterans, this travel burden was sometimes due to weather-related issues.

*It’s a little bit because of the snow and weather here in Montana, and the pass that I have to go over to get to the VA.*

#### Comfort With Technology

Several veterans did not have concerns regarding reduced quality of care with forgoing routine in-person visits and were comfortable with the quality of RM. As one veteran stated,

*The technology is going to continue to improve. And those monitors are just going to get better and better. So that really eliminates the need to go inside and talk to the technician… If I don’t have to [go to face-to-face visits], you’re not exposing yourself to other patients being sick and all that.*

Some veterans felt reassured that RM would adequately monitor their device.

*I think it would be alright as long as I know they’re checking my machine and make sure it’s up running.*

#### Reducing Burden on the Clinic

Some patients mentioned that this new model of care would reduce the burden on their VHA clinic, and help other veteran patients get care.

*Your clinician can actually be seeing somebody that’s really in need instead of doing a basic maintenance check.*

### Clinician Surveys

Of 22 clinicians interviewed, 20 (87%) participated in the survey, 14 (64%) of which were fully complete. Of the 20 respondents, 6 were MD/DOs, 7 were advanced practice providers (APPs), 6 were registered nurses (RNs), and 1 was a medical instrument technician (Table 3). Ten self-identified as female and 6 self-identified as non-White. Almost half of the respondents had been working at their current VHA cardiology clinic for >10 years. All clinicians were focused on CIED-related care and were not serving as patients’ primary cardiology clinician.

The most commonly reported scheduling frequency for routine in-person ICD and pacemaker evaluations was every 12 (range 4‐12) months, used by 72% (n=13) and 83% (n=15) of clinicians, respectively (Table 4).

Seven (39%) clinicians reported using an RM-first strategy for some patients. Sixteen (89%) thought this strategy would improve veteran convenience by reducing appointments and travel time. Six (33%) expected it would enable more care for other patients with heart rhythm disorders.

However, 12 (63%) clinicians were concerned about a reduction in the quality of veteran care and 10 (53%) about veteran-perceived abandonment. Fifteen (83%) respondents were confident that an RM-first strategy was as effective as RM with in-office visits regarding cardiovascular outcomes, while 3 (17%) were not. Seven (39%) expected an RM-first strategy would benefit their clinic, 7 (39%) were undecided, and 4 (22%) thought it would not.

**Table 3.** Clinician characteristics and perspectives on remote monitoring (RM)–first strategy.

Characteristic		Values, n (%)
Title (n=20)		
	Advanced practice provider	7 (35)
	Medical instrument technician	1 (5)
	Registered nurse	6 (30)
	Physician	6 (30)
Time worked with current VHA^a^ cardiology clinic (n=20)		
	<1 year	0 (0)
	1‐5 years	8 (40)
	6‐10 years	3 (15)
	>10 years	9 (45)
Adjustment to CIED^b^ care schedule if the patient does not want routine in-person CIED checks or misses an in-person check (n=19)^c^		
	Adjust the RM transmission schedule	3 (16)
	Reduce the frequency of in-person device checks	5 (26)
	Offer video visit paired with RM as an alternative	2 (11)
	Offer a telephone visit paired with RM as an alternative	9 (43)
	Other: encourage rescheduling an in-person visit	3 (16)
Current use of RM-first strategy for any patients (n=18)		
	Yes	7 (39)
	No	11 (61)
Benefits for RM-first strategy (n=18)^c^		
	Veteran convenience in reducing appointments and travel time	16 (89)
	Better use of clinic space	7 (39)
	Ability to see other patients with heart rhythm disorders	6 (33)
Concerns about an RM-first strategy (n=18)^c^		
	Changes to payment structure or relative value units	2 (11)
	Reduction in quality of veteran care	12 (63)
	Veteran patient impression of abandonment	10 (53)
	Reducing veteran contact with the VHA	9 (47)
Confidence that an RM-first strategy is as effective as RM + in-office evaluations for cardiovascular outcomes (n=18)		
	Not at all confident	3 (17)
	Somewhat confident	10 (56)
	Confident	3 (17)
	Somewhat more confident	1 (5)
	Very confident	1 (5)
Would an RM-first strategy help your clinic? (n=18)		
	Yes	7 (39)
	No	4 (22)
	Undecided	7 (39)

a VHA: Veterans Health Administration.

b CIED: cardiovascular implantable electronic device.

c Participants able to select multiple responses.

**Table 4.** Current frequency of routine in-person evaluations and remote transmission reviews reported by clinicians.

	For patients with implantable cardioverter-defibrillators, n (%)	For patients with pacemakers, n (%)
Frequency of routine in-person evaluation (n=18 clinicians)		
4 months	1 (6)	0 (0)
6 months	4 (22)	2 (11)
10 months	0 (0)	1 (6)
12 months	13 (72)	15 (83)
Frequency of transmission review without an in person visit (n=14 clinicians)		
3 months	4 (29)	4 (29)
5 months	1 (7)	0 (0)
10 months	0 (0)	1 (7)
12 months	1 (7)	1 (7)
Not applicable	8 (57)	8 (57)

### Clinician Interviews

#### Overview

Most interviewed clinicians were open to RM-first care, although some were not, and a few had no preference. Although many were hesitant, they still expected that RM-first care represented the future.

Many clinicians already had experience with RM-first care during the COVID-19 pandemic and noted that it reduced veteran travel time and clinician visit burden, but patient RM connectivity was a challenge. Most clinicians and facilities had returned to the prepandemic model of CIED care. Barriers and facilitators to RM-first care described by clinicians are in Table 2.

#### Barriers to RM-First Care

##### Lacking Routine In-Person Assessment

The most cited barrier by clinicians was that the benefits of routine in-person assessment during CIED clinic visits would not be available. These concerns ranged from a general sense that an in-person assessment was safer for patients, particularly for patients with greater complexity, such as those with advanced heart failure, to specifically valuing the physical examination and opportunity for in-person medication reconciliation. As a medical instrument technician stated,

*If we cannot assess their condition in-person, then we may find flags later that are really big issues and then we have to adjust everything.*

These concerns could also be related to missing important CIED information, including the occasional need for reprogramming.

##### Reducing Veteran Contact With VHA

Another clinician-cited barrier was that an RM-first approach would lead to a reduction in veteran contact with the VHA, which could potentially leave patients perceiving abandonment. As one RN stated,

*In-person visits are the expectation for many patients, so they could feel abandoned.*

A physician discussed the importance of the rapport built during routine in-person CIED visits,

*Face-to-face interactions with patients and doctors [are] important for rapport. Just putting your hand on them can make your relationship and their comfort with you better.*

Some clinicians expressed concern that patients would be lost to follow-up without in-person visits because device clinic visits are used to ensure that patients have other routine cardiology follow-up scheduled. As a physician stated,

*Patients always get lost to follow-up so it’s nice to have one more place to get eyes on them.*

##### Clinic Operations–Related Changes

Clinicians anticipated the need for operational changes to their clinic, including ensuring a reliable tracking system for patients not being seen in person to prevent patients from being lost to follow-up. As an APP stated,

*I don’t know that we have a system in place for the clinic as a whole to track things… between the device nurse, the provider and the EP nurse navigator [we would need] to develop some sort of tracking system.*

Clinicians also perceived a need for time to review more remote transmissions if patients were not receiving routine in-person device clinic evaluations. As an APP shared,

*Definitely more time on the nursing side to… get them [remote transmissions] processed into the charting system.*

Some felt that without an in-person visit, at least an annual review of the patient’s data would be important.

*I would still want a yearly review… I would go through it with a fine-toothed comb.*

Finally, there were concerns surrounding the loss of device clinician skills if patients were no longer routinely attending in-person visits, particularly for training new staff. As one RN shared,

*As self-taught on remote monitoring, we will get rusty on our skills… The learning curve is pretty steep… to feel comfortable to perform an interrogation independently. In-person clinic follow up is our only way of training… If we went remote-only, we would have no way of both training new staff and keeping current comfortable. Then when we would need to see patients, we would be at a severe disadvantage.*

##### Technological Difficulties

Interviewees noted that an RM-first approach placed increased importance on RM technology and some worried that veterans and clinicians may experience technological difficulties, particularly because RM adherence and connectivity were essential. As an RN stated,

*The tech is the stumbling block because it’s hard to troubleshoot the home monitor when it’s not working. Then you have to make them come in and some would not want to come after not coming for a while.*

### Benefits of RM-First Care

#### Reduced Veteran Travel Burden

Interviewees emphasized reduced veteran travel burden—including reduced travel time, cost, and weather-related issues. As an RN stated,

*[RM-first care] would be good for those patients who travel 200+ miles for 15-minute visits.*

Similarly, an electrophysiologist stated,

*Some drive more than 100 miles to get here... Winter storms are another example when it is dangerous to travel.*

An RN explained that some patients have difficulty arranging transportation and are unable to drive themselves to clinic visits,

*Some patients have 4 hours travel to our clinic…Staying home and only coming in for reprogramming needs would be useful. Cost has gone up as well, with fuel prices, being on the road and eating out. There are not great DAV transportation options. A lot of problems finding van drivers.*

Finally, a few clinicians thought that RM-first care may make some patients more likely to engage in CIED care. As one electrophysiologist noted,

*Some patients really turn off about having to come in. There are some who are more likely to engage through remote monitoring only.*

#### Optimization of Clinic Staff Time and Clinic Space

Another potential benefit of RM-first care was that it could optimize clinic staff time and often-limited outpatient clinic space. As 1 physician described,

*It would offload clinics, that’s [in-person CIED visits] a lot of work that APPs do. They could devote more time to a multitude of other tasks.*

The time could be used to evaluate other patients with heart rhythm disorders waiting for care, explained an APP,

*Downsizing device clinic space could increase in-person arrhythmia clinic space.*

#### Increased Time to Review Routine Remote Transmissions and Improve RM Adherence

Interviewees also mentioned that an RM-first care model could increase staff time to review routine remote transmissions and support RM adherence. One APP explained,

*Some of those remote transmissions are over 100 pages long. There are days when I get 10 or more device alerts and it takes time to go through EGMs (intracardiac electrograms) and not missing anything. It would provide more time on the nursing side.*

#### No Concern About Relative Value Unit Workload Credit

Finally, most clinicians thought there would be no issue with relative value units (RVUs) when transitioning to an RM-first model. As an RN said,

*No [concerns regarding RVUs]. ... Sometimes you get more RVUs reviewing patients’ remote transmissions. You can do a note for addressing a missed transmission. People need to know the benefit of reviewing more remote transmissions.*

### Implementation of RM-First Care

Clinicians thought that patients who were the best candidates for RM-first care were those without cardiac resynchronization therapy (CRT) devices who were adherent to RM, clinically stable and noncomplex, not device-dependent, not having frequent arrhythmias, good communicators, and facile with technology. One APP explained,

*There is a certain population that would be appropriate. Younger, less comorbidities, low pacing burdens, that sort of thing. Knowledgeable and familiar with RM.*

Many clinicians expected the decision about appropriateness for an RM-first strategy would initially be determined by the patient’s clinician, as an APP explained,

*Anyone that the provider deems appropriate. It will be joint decision-making between the patient and the provider. We will talk with them and assess what their goals are, and as long as they understand that based on remote monitoring they would still have to come into the clinic if clinically indicated.*

When asked how an RM-first care model should be evaluated for success, most clinicians thought patient satisfaction should be a key indicator, along with patient RM adherence. As an APP said,

*Adherence to remote monitoring. I think you would want adherence over 95%. How are the Vets feeling about it, are they satisfied? Surveys. A lot of Vets would be amenable.*

Respondents also thought it would be important to ensure there was no increase in adverse outcomes or rhythm issues not being identified promptly.

*Prove that there are no greater adverse cardiac outcomes. I will always be more conservative with my Veteran patients and wary of big changes in care.*

Respondents also discussed potential time savings with an RM-first approach. As an RN said,

*Measure time savings of remote monitoring.*

Many interviewees also noted that monitoring for missed RM transmissions would be central for a new RM-first care model, but most already had a process in place for doing so. One APP explained,

*We would follow the same scheduling tracking system we have now. It’s basically a log by manufacturer and when they were last seen.*

## Discussion

### Principal Results

The 2023 HRS expert consensus statement introduced “alert-based remote monitoring,” defined as “a combination of continuous connectivity with clinic visits that are prompted only by the detection of actionable events,” [2] which provides the basis for the RM-first care model that we discussed with veterans and clinicians. Both expressed interest in this model of CIED care and cited the benefit of reducing patient travel burden and enabling clinical bandwidth to care for other patients. However, patients sometimes preferred in-person evaluations (generally for non-CIED related medical reasons and the patient-clinician relationship), and some expressed concerns regarding technological issues with RM. Given the VHA’s central RM infrastructure that reviews all remote transmissions, VHA is well-positioned to implement and study this care model, which could inform other health systems and clinicians about the context of implementing RM-first care. Indeed, most clinicians expected that RM-first would ultimately become the standard of care for CIED management.

### Comparison With Prior Work

There is often substantial lag in implementing research and consensus recommendations into clinical practice, including inertia in initiating new care models [21,22]. Reasons for such inertia include overestimation of existing care as well as lack of practice organization to achieve therapeutic goals [22]. Providing patient and clinician education and support when implementing an RM-first care model will be important to overcome inertia, leverage facilitators, and surmount barriers.

### Strategies to Overcome Barriers in Implementation

Some patients worried about the quality of RM. To address this, patient-centered RM education should be provided before transitioning to RM-first care and emphasize to patients that any actionable findings on RM will prompt appropriate clinical actions, sometimes including in-person evaluations. Additionally, for patients to qualify for this care strategy, they need to be consistently and continuously connected to RM so clinically actionable events can be identified promptly. Thus, patients should be educated about ensuring RM connectivity and troubleshooting strategies based on their specific transmitter. Patients and clinicians also raised concerns regarding the loss of the in-person relationship and the inability to perform in-person assessment, such as a physical examination. To address this, device clinicians should ensure that patients have regular follow-ups with their general cardiologist or electrophysiologist (as appropriate) or at least routine primary care, and that the device clinic is not their primary source of cardiology care.

Clinicians also noted a potential increased risk of patients being lost to follow-up. Clinics must have a method of tracking patients outside of in-person visits and ensuring RM adherence [16]. Patients who become disconnected from RM will require in-person evaluation. Finally, patients and clinicians raised concerns about technical comfort with troubleshooting home monitors and RM adherence, which requires a high workload [23]. To alleviate this burden, postcard reminders that recommend patients contact their CIED manufacturer for assistance have been shown to increase RM adherence, without burdening clinicians [24]. Additionally, sending informational text messages to recently disconnected patients can improve RM adherence [25].

### Benefits of Implementation

Although there are several barriers to be addressed, the RM-first care model has the potential to provide many improvements for patients and clinicians. With the growing potential of digital health technology in cardiovascular medicine [26], the lessons from our study have broad applicability but it will be critical to ensure that an RM-first care model, as with any virtual care modality, is implemented equitably [27,28]. Reduced patient travel burden is particularly important for patients who live in rural locations. From a reimbursement perspective, while VHA is a single-payer, other health care payers would need to adopt novel reimbursement strategies for RM that facilitate sustainable and cost-effective CIED follow-up care [2,29,30]. Finally, a reduction in unnecessary device-related clinic visits will allow clinicians to see other patients with heart rhythm disorders and reduce wait times, which may result in higher-value care, particularly given the shortage of cardiovascular health professionals [31]. An RM-only model has been successfully implemented at a large clinic in Italy since the COVID-19 pandemic and was associated with time savings for clinicians and patients with no increase in adverse clinical outcomes [32]. Further, although not currently available, if remote reprogramming is demonstrated to be safe and feasible to implement, it could further reduce the need for in-person visits and could improve patient perceptions around an RM-first care model.

### Limitations

Our study should be considered in the context of its limitations. First, although we studied a single health system with specific patient population demographics (more often rural, predominantly White, and predominantly male) and clinicians providing care in an integrated health care delivery system, the Veterans Affairs National Cardiac Device Surveillance Program (VANCDSP) centrally monitors more than 64,000 veterans with CIEDs, making VHA well-positioned to implement and evaluate RM-first care. Future studies should evaluate other patient populations, which would help to assess the transferability of our findings. Second, although this was a national study, our results represent a limited number of both patient and clinician perspectives. However, qualitative methods intentionally provide granular data from smaller numbers of participants, patients were randomly selected, and our methodology provided detailed information on perspectives from clinicians across the United States. Third, interviews were conducted while new HRS consensus was released in draft form [2], so questions were modified partway through the interview process, and the ideas being introduced were new; patients and clinicians may feel differently when they have had more time to assimilate the recommendations. We did not inform patients about the additional safety offered by consistent and continuous RM connectivity. Fourth, we did not interview patients who were new or nonadherent to RM. Fifth, we did not have participant validation of our findings. Sixth, this study’s team represented an institution (VANCDSP) with some influence on both patient care and clinical support. While it was not apparent in the review of interview recordings or transcripts, this power dynamic may have incentivized veteran patients and clinicians to speak more favorably of the VANCDSP or caused interviewees to present their care or their patient’s existing care in a more favorable light. Finally, this study represents patient and clinician expectations of RM-first care, instead of their views based on experience; as RM-first is implemented in the future, patient and clinician perceptions on barriers and facilitators to this care model should be evaluated.

### Conclusions

Both patients and CIED clinicians experienced in RM within the VHA were open to an RM-first care model that reduces in-person visits but conveyed barriers about solely relying on RM and possible diminution of the patient-clinician relationship. Implementation of new RM recommendations will require attention to these perceptions and prioritization of patient-centered approaches.

## Supplementary material

10.2196/66215 Multimedia Appendix 1 Final clinician and veteran interview guides and clinician survey.

## Abbreviations

APP: advanced practice provider
CIED: cardiovascular implantable electronic device
HRS: Heart Rhythm Society
ICD: implantable cardioverter-defibrillator
RM: remote monitoring
RN: registered nurse
RVU: relative value unit
VA: Veterans Affairs
VANCDSP: Veterans Affairs National Cardiac Device Surveillance Program
VHA: Veterans Health Administration
