# Supplementary material for: Patient and Clinician Perspectives on Alert-Based Remote Monitoring–First Care for Cardiovascular Implantable Electronic Devices: Semistructured Interview Study Within the Veterans Health Administration
Source: JMIR Cardio. 2025 Apr 4;9:e66215. doi: 10.2196/66215 (PMC11990655; doi:10.2196/66215)
Supplement: Multimedia Appendix 1 [file cardio-v9-e66215-s001.docx]

# **Final Interview Guide for Veterans Health Administration Cardiology Device Clinicians**

Bolded questions below are those which are reported in the manuscript.

## Introduction

Thank you again for taking time out of your busy schedule to speak with us.

Before we get started, I wanted to give you some more background on the project and then see if you have any questions. This quality improvement study is being conducted in collaboration between the VA Office of Rural Health, National Cardiac Device Surveillance Program (NCDSP), and Measurement Science QUERI, with the goal of improving access and quality of care for rural Veterans with pacemakers and implantable cardioverter-defibrillators by reducing avoidable in-person visits in favor of virtual care for rural Veterans. In addition to this interview with you and other cardiology device clinic leadership around the country about the clinical aspects of potentially altering their care strategies, we are interviewing veterans living in rural areas to understand their perspectives on reducing these avoidable visits.

With your permission, we will be recording the audio from this interview and using the Teams transcription feature to keep a record for analysis as well as taking notes. As a reminder, you can skip any question that you don’t want to answer. However, your identity will be kept confidential and nothing you say will be able to be traced back to you. The interviews can last up to an hour – does that still work with your schedule?

One last bit of background before we jump in: During the interview, we will refer to a virtual-first care strategy. In this care model, selected veterans with CIEDs (that is, Pacemakers and ICDs) are seen through virtual visits (by which we mean telephone or VVC), with in-person visits only if clinically indicated (because of abnormalities on a remote transmission or non-adherence to remote monitoring), as opposed to routinely scheduled. Veteran’s other clinical care, including primary care and general cardiology care, would continue as usual.

We will also refer to a Remote Monitoring Only strategy, where patients who are adherent to monitoring (defined as having sent at least one transmission in the past 100 days) would not be scheduled for a virtual or in-person visit unless clinically indicated or if they become non-adherent. Two randomized trials in non-VA populations with CIEDs comparing remote monitoring only vs remote monitoring + in-office evaluations^1,2^ have shown no difference in cardiovascular outcomes. Additionally, the 2023 Draft Heart Rhythm Society Expert Consensus Statement on Practical Management of the Remote Device Clinic suggests that because most visits conclude that the CIED is working properly and no further action is needed, one could argue that that they may be considered “low value” and that patients with continuous CIED monitoring may be able to transition to alert-based care, with visits based only on device alerts that suggest device reprogramming or other intervention is needed for patient care.

[Share the table via screenshare for further clarification]

**Patient Care Strategies for Veteran Patients with CIEDs Who are Clinically Stable**

|  | Routine In-Person Visits | Virtual-First Care | Remote Monitoring Only |
| --- | --- | --- | --- |
| In-person visits | Routinely scheduled | Only if clinically indicated* | Only if clinically indicated* |
| Scheduled virtual appointments (telephone, VVC) | None | Routinely scheduled | Only if clinically indicated* |
| Remote monitoring adherence (at least 1 transmission every 100-days) | Very important | Necessary | Necessary |
| Effect on other clinical care (primary care and general cardiology care) | No change | No change | No change |

*Clinical indications for in-person or virtual visits include abnormalities on a remote transmission, need for reprogramming, or non-adherence to remote monitoring.

Do you have any questions for me before we get started? Are our definitions/explanations of these two approaches clear to you?

Okay, I am turning on the recording.

## Part 1: Virtual Care with COVID-19 and Beyond

We’d first like to ask you to think back to early 2020, when the COVID-19 pandemic led VA to reduce routine in-person visits.

- **How did the transition to virtual care for the management of CIEDs go?**
  - What were the main challenges?
  - What are your main success stories?
- **How has your approach to CIED management for clinically stable patients changed since the start of the COVID-19 Pandemic?**
  - Have there been any other big changes to virtual care since 2020?
- Apart from following up with patients when an alert or abnormal transmission requires, does your clinic use virtual care visits for routine CIED management?
  - What are the main advantages/successes?
  - What are the main disadvantages/challenges?

For management of patients with CIEDs, understanding if a patient missed a remote transmission is absolutely crucial for both the remote monitoring only and virtual-first strategies.

- How do you track or how would you like to track adherence to remote monitoring if a patient is “virtual-first” or “RM-only”?

*Probes: How would you address a missed remote transmission if a patient is “virtual-first” or “RM-only”? Describe your system for tracking remote monitoring.*

- Would you distinguish your adherence efforts between continuous connectivity vs the standard scheduled transmission (generally every 12 weeks or 90 days)?
- What is your care model for patients with implantable loop recorders (ILRs)/implantable cardiac monitors (ICMs)? Do patients have any routine in-person visits?

## Part 2: Remote Monitoring Only Strategy

**Next, we would like to talk about your impressions of the remote monitoring only strategy moving forward.**

- **What would be the primary benefits of a remote monitoring only approach?**
- **What is your biggest concern with the remote monitoring only approach?**
- **Describe the kind of patient for whom a remote monitoring only strategy would be most successful and why.**

*Probes: Patients with CIEDs who live especially far from your clinic? Patients with primary prevention ICDs? Pacemaker patients who are not dependent on the pacemaker? Patients with high adherence to remote monitoring?*

- **Describe the kind of patient for whom a remote monitoring only strategy would not be appropriate and why.**

*Probes: patients with cardiac resynchronization therapy devices? Patients with low adherence to remote monitoring?*

- **Do you foresee a future where the standard of care for routine follow-up of patients with CIEDs is a remote monitoring only strategy? Why or why not?**

**We’d now like to discuss how shifting to a remote monitoring only strategy for CIED care may affect the workflows for your clinic:**

- **How would a remote monitoring only approach affect your overall clinic workflow?**

*Probes: Time to devote to other tasks? What tasks?*

- How would you reorganize your clinic to schedule only as-needed in-person evaluations (as opposed to routine in-person evaluations) for CIED checks?

*Probes: shift in the roles of some staff members?*

- **What concerns do you have about transitioning to remote monitoring only instead of in-person or virtual visits?** [reference survey responses]

*Probes: shift of relative value units (RVUs)?*

Part 2.1 Q2 year Visits:

- There is a Draft Heart Rhythm Society Expert Consensus Statement, released in December 2022, which has a Class 2A recommendation that for patients with Pacemakers and ICDs on RM with consistent and continuous connectivity, and in the absence of recent alerts or other cardiac comorbidity, it is reasonable to schedule in-person visits every 24 months.
- Do you plan to transition any patients to this 2-year follow-up schedule if the document is finalized? Why or why not?
- The Draft Statement also suggested that the ideal model of care may actually be an alert-based strategy. In this care model, patients would have no routine in-person visits and no routine remote transmissions. They would be continuously and consistently connected, but would not send remote transmissions unless they triggered an alert.
- **What are your thoughts on this strategy?**
- **Do you think that this is a feasible model of care? Why are why not?**

*Probes: Do you have concerns about tracking continuous connectivity?*

Part 3: Virtual-First Care

Next, we would like to talk about your impressions of the virtual-first care strategy moving forward.

- **What would be the primary benefits of a virtual-first approach?**
- **What is your biggest concern with the virtual-first strategy?**
- Describe the kind of patient for whom a virtual-first strategy would be most successful and why.
- Describe the kind of patient for whom a virtual-first strategy would not be appropriate and why.
- Do you expect the standard of care for routine follow-up of patients with CIEDs to shift to a virtual-first strategy? Why or why not?

Next, we’d like to ask about how a virtual first strategy would impact your clinic:

- How would a virtual-first approach affect your overall clinic workflow?

*Probes: Time to devote to other tasks? What tasks?*

- What concerns do you have about transitioning to virtual instead of in-person visits? [reference survey responses]

*Probes: shift of relative value units (RVUs)?*

## Part 4: Follow-up

- Who within your clinic could help champion or is already advocating for a transition to a virtual-first strategy or remote monitoring only strategy of care for Veteran patients with CIEDs?
- Who within your clinic would be able to make the decision to transition to a virtual-first strategy or remote monitoring only strategy of care for clinically stable Veteran patients with CIEDs?
- What resources and support would your clinic need to move towards a remote monitoring only strategy for Veteran patients with CIEDs?
- How can the process of implementation be evaluated?

*Probes: What would define a successful virtual-first care delivery strategy?*

*What would define a successful remote monitoring only strategy?*

# **Survey for VA Cardiology Device Clinicians**

**Title:** Pre-Interview Survey – Virtual Care for Veterans with CIEDs

**Description:** This survey asks about your experience with virtual care appointments for veterans who have a CIED (cardiac implantable devices including implantable cardiac devices or pacemakers) and your perspectives about reviewing transmissions without a patient visit.

## Professional Background

XX. **Please tell us your title within your VA clinic**

- **DO/MD**
- **Advanced Practice Provider (APP)**
- **RN**
- **Medical Instrument Technician (MIT)**
- **Other**

XX. **How many years have you been working with your current VA cardiology clinic?**

- **<1 year**
- **1-5 years**
- **6-10 years**
- **>10 years**

## CIED In-Person Checks

**How often (on average) do you conduct routine evaluations for Veteran patients with CIEDs?**

|  | **Adherent patients** | **Non-adherent patients (i.e. no remote transmission in past 100 days)** |
| --- | --- | --- |
| **In person evaluation of patient with ICD** | **Drop down list of 1-12 months or N/A** | **Drop down list of 1-12 months**  **or N/A** |
| **In Person evaluation of patient with pacemaker** | **Drop down list of 1-12 months**  **or N/A** | **Drop down list of 1-12 months**  **or N/A** |
| Virtual (phone or VVC) evaluation of patient with ICD | Drop down list of 1-12 months  or N/A | Drop down list of 1-12 months  or N/A |
| Virtual (phone or VVC) evaluation of patient with pacemaker | Drop down list of 1-12 months  or N/A | Drop down list of 1-12 months  or N/A |
| **Transmission review (without visit) of patient with ICD** | **Drop down list of 1-12 months**  **or N/A** | **Drop down list of 1-12 months**  **or N/A** |
| **Transmission review (without visit) of patient with pacemaker** | **Drop down list of 1-12 months**  **or N/A** | **Drop down list of 1-12 months**  **or N/A** |

XX. **If a patient does not want to schedule routine in-person CIED checks or misses an in-person CIED check, how do you adjust their CIED care schedule?**

- **Adjust remote monitoring transmission schedule**
- **Reduce frequency of in-person device checks**
- **Offer VA Video Connect (VVC), paired with a remote transmission, as an alternative**
- **Offer telephone visit, paired with a remote transmission, as an alternative**
- **Other (please specify)**

<select all that apply>

## Perspectives on virtual-first care

Description: A virtual-first care strategy is a care delivery model where Veterans with CIEDs (that is, Pacemakers and ICDs) are seen through virtual visits (i.e., telephone or VVC), with in-person visits only if clinically indicated, as opposed to routinely scheduled. Veteran’s other clinical care, including primary care and general cardiology care, would continue as usual.

XX. Are you currently using a virtual-first strategy for any patients?

<yes/no>

XX. Which of the following do you believe are benefits of a virtual-first strategy vs. routine in-person visits?

- Veteran convenience in reducing travel to clinic
- Better use of clinic space
- Ability to see more patients
- Other (Fill in the Blank)

<Mark all that apply>

XX. Please select concerns that you have with a virtual-first care strategy.

- Changes to payment structure/RVUs
- Reduction in quality of veteran care
- Quality of VVC or telephone connection
- Ability for veterans to use VVC or participate in telephone calls
- Reducing veteran contact with VA
- Other (fill in the blank)

<Mark all that apply>

XX. How confident are you that a virtual first strategy (with in-person visits as needed) is as effective as remote monitoring + in-office evaluations in terms of cardiovascular outcomes

- <likert scale: Not at all confident, somewhat less confident, confident, somewhat more confident, very confident>

XX. How much of a transition would your clinic need in order to become a virtual-first clinic?

- <likert scale: A lot of change, some change, very little change, no change – we are a virtual-first clinic>

XX. Overall, would a virtual-first strategy help your clinic?

- <yes/no/undecided>
- <fill in the blank>

## Perspectives on remote monitoring only care

**Description:** **Perspectives on remote monitoring only care**

**A remote monitoring only strategy** is a care delivery model where Veterans with CIEDs (that is, Pacemakers and ICDs) who are adherent to remote monitoring are scheduled for a **visit (virtual visits or in-person) only if clinically indicated, as opposed to routinely scheduled**. Veteran’s other clinical care, including primary care and general cardiology care, would continue as usual.

Two randomized trials in non-VA populations with CIEDs comparing remote monitoring only vs remote monitoring + in-office evaluations have shown no difference in cardiovascular outcomes (Garcia-Fernandez et al, 2019 <https://doi.org/10.1093/eurheartj/ehz067>; Watanabe et al, 2020 <https://doi.org/10.1161%2FCIRCEP.119.007734>).

|  | Routine In-Person Visits | Virtual-First Care | Remote Monitoring Only |
| --- | --- | --- | --- |
| In-person visits | Routinely scheduled | Only if clinically indicated* | Only if clinically indicated* |
| Scheduled virtual appointments (telephone, VVC) | None | Routinely scheduled | Only if clinically indicated* |
| Remote monitoring adherence (at least 1 transmission every 100-days) | Very important | Necessary | Necessary |
| Effect on other clinical care (primary care and general cardiology care) | No change | No change | No change |

XX. **Are you currently using a remote monitoring only strategy for any patients?**

**<yes/no>**

XX. **Which of the following do you believe are benefits of a remote monitoring only strategy vs. routine virtual care or in-person visits?**

- **Veteran convenience in reducing appointments and travel time**
- **Better use of clinic space**
- **Ability to see other patients with heart rhythm disorders**
- **Other (Fill in the Blank)**

<Mark all that apply>

XX. **Please select concerns you have with a remote monitoring only strategy.**

- **Changes to payment structure/RVUs**
- **Reduction in quality of veteran care**
- **Veteran patient impression of abandonment**
- **Reducing veteran contact with VA**
- **Other (fill in the blank)**

<Mark all that apply>

XX. **How confident are you that remote monitoring only (with virtual or in-person visits only as needed) is as effective as remote monitoring + in-office evaluations with respect to cardiovascular outcomes?**

**<likert scale: Not at all confident, somewhat less confident, confident, somewhat more confident, very confident>**

XX. **Overall, would a remote monitoring only strategy help your clinic?**

**<yes/no/undecided>**

If you answered yes or no to the above question, why or why not?

<fill in the blank>

XX. List any local initiatives focused on virtual care into which you think a virtual-first or remote monitoring only strategy fit in?

<fill in the blank>

## Demographic Questions

XX. Please provide your age (years).

- <30
- 31-40
- 41-50
- 51-60
- 61-70
- 71 or older

<select one>

XX. How many years have you worked within VA?

- <1 year
- 1-5 years
- 6-10 years
- >10 years

<select one>

XX. How many years have you worked in CIED care or management?

- <1 year
- 1-5 years
- 6-10 years
- >10 years

<select one>

XX. Please provide your gender self-identification

- Woman
- Man
- Transgender woman
- Transgender man
- Non-binary
- Other
- Prefer not to say

<select one>

XX. Please provide your racial and ethnic self-identification

- American Indian or Native American
- Asian
- Black or African American
- Hispanic, Latinx, or of Spanish origin
- Pacific Islander
- White
- Other
- Prefer not to say

<select all that apply>

XX. Are there any other individuals at your device clinic who are focused on care of Veterans patients with CIEDs with whom you think we should speak? If so, please provide their email address.

<Text box>

XX. Additional Comments

<text box>

# **References**

1. Garcia-Fernandez FJ, Osca Asensi J, Romero R, et al. Safety and efficiency of a common and simplified protocol for pacemaker and defibrillator surveillance based on remote monitoring only: a long-term randomized trial (RM-ALONE). Eur Heart J. 2019;40(23):1837-1846.
2. Watanabe E, Yamazaki F, Goto T, et al. Remote Management of Pacemaker Patients With Biennial In-Clinic Evaluation: Continuous Home Monitoring in the Japanese At-Home Study: A Randomized Clinical Trial. Circ Arrhythm Electrophysiol. 2020;13(5):e007734.

#

# **Final Interview Guide for Rural Veterans**

**Background:**

[Veteran Name], before we get started, I wanted to give you some more background on the project and then see if you have any questions.

This study is a collaboration between the VA Office of Rural Health, the National Cardiac Device Surveillance Program (NCDSP), and Measurement Science QUERI. The goal is to improve access and quality of care for Veterans living in rural areas who have pacemakers and implantable defibrillators. We want to try do this by reducing avoidable in-person visits in favor of virtual care for rural Veterans.

**Recording Permission**:

With your permission, we will be recording the audio from this interview. We will then transcribe the recording and use the information for analysis. However, your identity will be kept confidential and nothing you say will be able to be traced back to you. Also, you can skip any question that you don’t want to answer. Just let me know and we’ll go on to the next question. We hope to get the interview done in 15 to 20 minutes, but may take a bit longer, depending– does that still work with your schedule? Do you have any questions for me before we get started?

**No To Recording:**

I see, would you rather not do the interview, or could I try to just write down some notes instead of the recording? It may take a bit longer though.

**Start Recording**

Okay, I am turning on the recording. I won’t use your name from here on in, so I hope you’ll forgive the informality….

**Begin Interview:**

1. We’d first like to ask you to think back to early 2020, back when the COVID-19 pandemic first led to a reduction in in-person appointments at VA.

- So, during the COVID-19 pandemic, when things changed to primarily virtual-only care and nearly all in-person appointments stopped, what, if any, challenges did you encounter related to your pacemaker/ICD? Did the COVID pandemic cause any problems for you and your implant care?
- What, if anything, as related to the care of your pacemaker/ICD was improved?

*For example:* *Did you start doing* *remote care and wind-up saving* *travel time, or not having to come into the VA for care? (Travel burden?)*

*A.1)* ***How long to get to local VA? How do you get there?***

***Do you bundle your visits? Just cardiac or other non-cardiac care.***

***(Do you bundle your cardiac care visits with other visits).***

***Does it exhaust you to travel so much? Do you generally travel to the VA alone or with someone else like a family member or friend?***

1. Now we would like to discuss providing you with the option of reducing in-person visits for the care of your pacemaker/ICD. Specifically, instead of having to come into your local VA every 3 to 12 months to have your pacemaker/ICD checked, you would have the opportunity to have your pacemaker/ICD monitored electronically only without regularly scheduled appointments. Now this would not affect any of your other visits, such as with your cardiologist or primary care clinician. This is only for the in-person visits for your pacemaker/ICD.

It is important to remember that your VA clinicians receive the same data through remote/home monitoring as at an in-person visit. If there was an abnormality on your remote/home monitoring transmission, then you may have to come in for an in-person visit, as needed. You would still be able to contact your cardiology device clinic for any reason. Rigorously conducted studies have shown that electronic only monitoring is just as safe as having routine in-person visits.

**Electronic Only Monitoring**

- **Would you like the option of enrolling in an electronic only monitoring? You would still attend VA appointments that are unrelated to your pacemaker/defibrillator, but, unless you experienced any problems, your pacemaker remote monitoring would be done electronically. Why or why not?**
- **How would switching to electronic only monitoring for your pacemaker/ICD affect you? Are there ways in which this would be better for you? Are there any drawbacks that you may foresee?**
- **How do you think that switching to electronic only monitoring would affect the quality of care for your pacemaker/ICD?**

*Probes: how do you think it would specifically affect clinicians caring other clinical issues for you?*

- Would switching to electronic only monitoring change the importance that you place on your remote monitoring transmissions? Why or why not?
- If your transmitter is wireless, would switching to electronic only monitoring change the importance that you place on making sure that your transmitter is set up properly and transmitting? Why or why not?
- What, if anything, would you need to help you switch to electronic only monitoring for your pacemaker/ICD?

*Probes: information that this approach is safe? Feedback from clinicians about results of remote monitoring transmissions? Information about how to contact cardiology clinicians about pacemaker/ICD if not having routine in-person visits for your pacemaker/ICD?*

- **Would you need feedback from VA that remote transmission went through?**

**HRS Draft Recommendation**

- There is a new draft recommendation from the professional society of heart rhythm clinicians to monitor your pacemaker/ICD electronically and extend the time between in-person visits to 2 years. The doctor would contact you if there were any problems in the meantime.
- **Would you like the option of extending the time between in-person visits to 2 years? Why or why not?**

**Virtual-First Option**

Now we would like to discuss providing you with the option of reducing in-person visits for the ***care of your pacemaker/ICD*** but maintaining virtual visits, in addition to electronic monitoring. Specifically, instead of having to come into your local VA every 3 to 12 months to have your pacemaker/ICD checked, you would have the opportunity to substitute those visits with either a telephone or video visit. That, paired with the electronic monitoring that you are already doing. Again, this would not affect any of your other visits, such as with your cardiologist or primary care clinician.

So, my question is:

- Have you used virtual visits instead of in-person visits before, for your pacemaker/ICD?

**Yes to Previous Virtual Visits**

- Have the virtual visits been a telephone call? A video visit? Both?
- How often do have virtual visits? Are they regularly scheduled visits or are they special calls because of an adverse event reported to your cardiologist?
- What do you like about having a virtual visit instead of an in-person visit?

*Probes:* *travel time? Travel burden? Face to face connection with VA cardiology clinicians?*

- What do you dislike about having a virtual visit instead of an in-person visit?
- How has reducing in-person visits for your pacemaker/ICD affected you?

*Probes: How has it specifically affected your clinicians?*

- Has reducing in-person visits increased the importance that you place on your remote monitoring transmissions? Why or why not?

**Wrap Up**

OK, I think that is about all we needed to go over. Thank you so much for your answers.

Did you have any questions before we wrap it up?

Great! Thanks again and have a great day!

**No to Previous Virtual Visits**

- Would you like the option of replacing routine in-person visits to have your pacemaker/ICD checked with virtual visits (by which we mean that you would see your device clinicians through a video or telephone visit) instead? Why or why not?

*Probes:* *travel time? Travel burden? Face to face connection with VA cardiology clinicians?*

- How would reducing in-person visits for your pacemaker/ICD affect you?
- How do you think that reducing routine in-person visits would affect the quality of care for your pacemaker/ICD?

*Probes: how do you think it would specifically affect clinicians caring for other clinical issues for you?*

- Would reducing routine in-person visits increase the importance that you place on your remote monitoring transmissions? Why or why not?
- What would you need, if anything, to help you reduce routine in-person visits for your pacemaker/ICD?

*Probes: information that this approach is safe? Feedback from clinicians about results of remote monitoring transmissions? Information about how to contact cardiology clinicians about pacemaker/ICD if not having routine in-person visits?*

**Wrap Up**

OK, I think that is about all we needed to go over. Thank you so much for your answers. Did you have any questions before we wrap it up?
